# Autophagy Controls BCG-Induced Trained Immunity and the Response to Intravesical BCG Therapy for Bladder Cancer

**DOI:** 10.1371/journal.ppat.1004485

**Published:** 2014-10-30

**Authors:** Kathrin Buffen, Marije Oosting, Jessica Quintin, Aylwin Ng, Johanneke Kleinnijenhuis, Vinod Kumar, Esther van de Vosse, Cisca Wijmenga, Reinout van Crevel, Egbert Oosterwijk, Anne J. Grotenhuis, Sita H. Vermeulen, Lambertus A. Kiemeney, Frank L. van de Veerdonk, Georgios Chamilos, Ramnik J. Xavier, Jos W. M. van der Meer, Mihai G. Netea, Leo A. B. Joosten

**Affiliations:** 1 Department of Internal Medicine, Radboud University Medical Center, Nijmegen, The Netherlands; 2 Radboud Institute of Molecular Life Sciences (RIMLS), Radboud University Medical Center, Nijmegen, The Netherlands; 3 The Broad Institute of Massachusetts Institute of Technology and Harvard University, Cambridge, Massachusetts, United States of America; 4 Center for Computational and Integrative Biology and Gastrointestinal Unit, Massachusetts General Hospital, Harvard Medical School, Boston, Massachusetts, United States of America; 5 University of Groningen, University Medical Center Groningen, Department of Genetics, Groningen, The Netherlands; 6 Department of Infectious Diseases, Leiden University Medical Center, Leiden, The Netherlands; 7 Department of Urology, Radboud University Medical Center, Nijmegen, The Netherlands; 8 Department for Health Evidence, Radboud University Medical Center, Nijmegen, The Netherlands; 9 Radboud Institute of Health Sciences (RIHS), Radboud University Medical Center, Nijmegen, The Netherlands; 10 Department of Human Genetics, Radboud University Medical Center, Nijmegen, The Netherlands; 11 Department of Medicine, University of Crete, Heraklion, Crete, Greece; University of New Mexico, United States of America

## Abstract

The anti-tuberculosis-vaccine Bacillus Calmette-Guérin (BCG) is the most widely used vaccine in the world. In addition to its effects against tuberculosis, BCG vaccination also induces non-specific beneficial effects against certain forms of malignancy and against infections with unrelated pathogens. It has been recently proposed that the non-specific effects of BCG are mediated through epigenetic reprogramming of monocytes, a process called *trained immunity*. In the present study we demonstrate that autophagy contributes to trained immunity induced by BCG. Pharmacologic inhibition of autophagy blocked trained immunity induced *in vitro* by stimuli such as β–glucans or BCG. Single nucleotide polymorphisms (SNPs) in the autophagy genes *ATG2B* (rs3759601) and *ATG5* (rs2245214) influenced both the *in vitro* and *in vivo* training effect of BCG upon restimulation with unrelated bacterial or fungal stimuli. Furthermore, pharmacologic or genetic inhibition of autophagy blocked epigenetic reprogramming of monocytes at the level of H3K4 trimethylation. Finally, we demonstrate that rs3759601 in *ATG2B* correlates with progression and recurrence of bladder cancer after BCG intravesical instillation therapy. These findings identify a key role of autophagy for the nonspecific protective effects of BCG.

## Author Summary

Next to its effects against tuberculosis, BCG vaccination also induces non-specific beneficial effects on immune cells to increase their ability to control unrelated pathogens. It has been recently proposed that the non-specific effects of BCG are mediated through epigenetic reprogramming of monocytes, a process called trained immunity. Little is known regarding the intracellular events controlling its induction. In this study we identified autophagy as a key player in trained immunity. Pharmacological inhibition of autophagy as well as polymorphisms in autophagy-related genes blocked BCG-induced trained immunity. Furthermore, BCG vaccine is also used to treat bladder cancer. Genetic polymorphisms in autophagy-related genes correlated with progression and recurrence of bladder cancer after treatment with BCG therapy. These findings open new possibilities for improvement of future BCG-based vaccines to be used against infections and malignancies.

## Introduction

Immunological memory has long been viewed as being exclusively mediated by T and B cells. However, an increasing body of evidence indicates enhanced nonspecific protection against reinfections in plants [1] and insects [2] which lack adaptive immunity. Similarly, mammalian innate immune cells such as natural killer cells show features of immunological memory [3], [4]. Recently, we proposed the term *trained immunity* to describe the memory properties of innate immune cells [5]. *Candida albicans* or its major cell wall component β-glucan, as well as BCG, are prominent stimuli that can induce trained immunity through epigenetic reprogramming of monocytes [6], [7]. However, little is known regarding the intracellular events controlling the induction of trained immunity, impairing the ability to fully harness the therapeutic potential of this important immunological process. Therefore, we investigated the trained immunity-induced signaling pathways, discovering autophagy being one of the main players.

## Results

### β-glucan training induces the transcription of autophagy-related proteins

**Figure 1:**
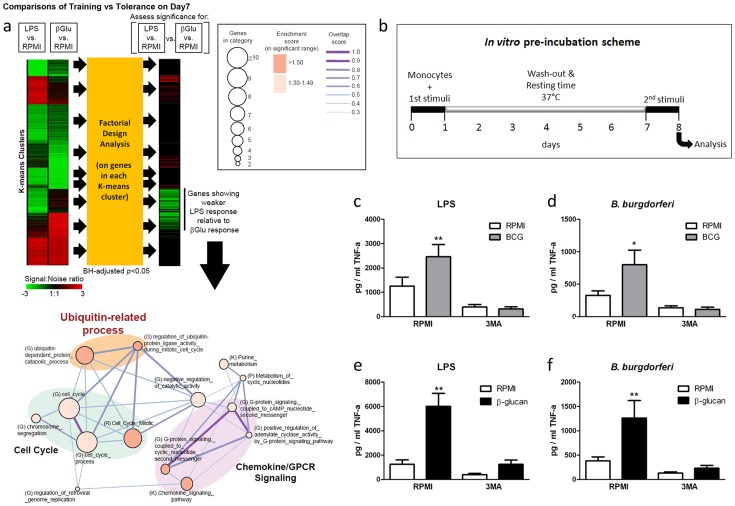
Role of autophagy for the training of monocytes. (a) Transcriptome profiling and pathway analysis of β-glucan training of monocytes compared to LPS stimulation. Factorial design analysis was performed on genes in each K-means cluster to assess significance of response differences elicited by LPS and β-glucan (Benjamini-Hochberg (BH)-adjusted *p*<0.05). The signal∶noise ratio is shown as heatmaps. Functional enrichment (or molecular concept) map was generated for genes exhibiting significantly weaker LPS response relative to β-glucan response. This map summarizes the extent of mutual overlap between gene sets and identifies a cluster of strongly connected gene sets that are enriched among genes showing stronger β-glucan response. Only enriched gene sets in the significant range with gene set enrichment score (−Log_10_(*p*)>1.3; *p*<0.05) are shown. Nodes denote enriched gene sets or “annotation terms/categories”, assembled from (K) KEGG pathways, (G) Gene Ontology, (P) Panther pathways, (R) Reactome. Node size corresponds to the number of gene members in each gene set. Node color denotes the gene set enrichment score. Please refer to graphical legend (boxed) in figure. The extent of mutually overlapping genes between gene sets is represented by thickness and color intensity of edges connecting nodes. The overlap score is the average of the Jaccard and Overlap coefficients. Strongly connected network components were identified using Tarjan's algorithm. Important ubiquitin-related processes in map are highlighted. (b) Diagram showing the course of the *in vitro* preincubation experiment. (c–f) BCG (c–d) or β-glucan (e–f) training *in vitro* in the presence or absence of 3MA using freshly isolated human monocytes and different stimuli for restimulation (LPS, *B. burgdorferi*). *P<0.05, **P<0.01.

To identify new signaling pathways specifically activated upon training of monocytes with bacterial components, we compared the transcriptional profile of β-glucan-trained human primary monocytes isolated from healthy volunteers to the profile of monocytes stimulated with *Escherichia coli*-derived lipopolysaccharide (LPS), which stimulates inflammation but is unable to induce long-term training [5]. Transcriptomic assessment of these monocytes by microarrays and pathway analysis revealed specific clusters of genes significantly induced by β-glucan training with an intriguing signal found in the ubiquitin-related proteins and associated catabolic processes (Figure 1a). Since ubiquitination plays an important role in autophagy [8], a process that has previously been shown to improve intracellular processing of BCG [9], [10], we examined the role of autophagy in the induction of trained immunity.

### Autophagy is essential for β-glucan and BCG training in monocytes

Using an *in vitro* model of trained immunity [6], [7], adherent monocytes from healthy human volunteers were stimulated for 24 h with RPMI, BCG or β-glucan alone or in combination with the autophagy inhibitors 3-methyladenine (3MA) or wortmannin. After washing of cells and a resting period of 6 days in medium supplemented with 10% human serum, cytokine production was measured after a second stimulation with the unrelated stimuli LPS or *Borrelia burgdorferi* (*B. burgdorferi*) (Figure 1b). IL-6 and TNF-α production increased significantly in BCG- and β-glucan-trained cells compared to non-trained cells. When autophagy was blocked by 3MA or wortmannin, neither β–glucan nor BCG induced *trained immunity* (Figure 1c–f; Figure S1a–h). Notably, the putative cytotoxic effects of autophagy inhibitors used in this study were assessed by LDH measurements. None of the inhibitors used during the 24 h of primary cell stimulation enhanced LDH release compared to RPMI-treated cells (Figure S2 a–c), demonstrating that the molecules were not toxic to the cells.

### Single nucleotide polymorphisms in *ATG2B* and *ATG5* negatively influence trained immunity

**Figure 2:**
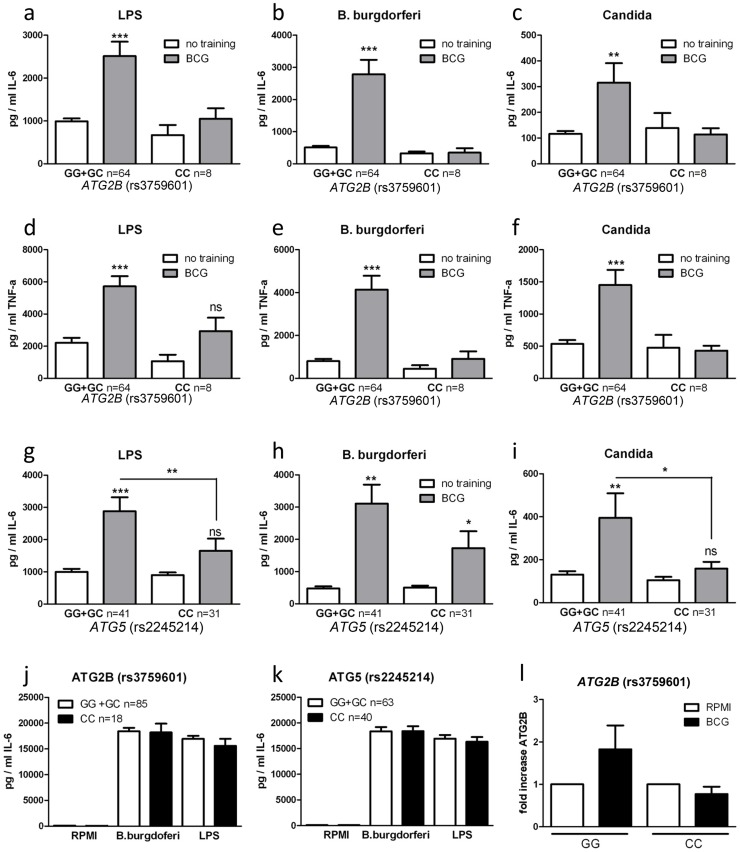
Polymorphisms in *ATG2B* or *ATG5* diminish the training capacity of human monocytes. (a–i) Blood was collected from volunteers and genotyped for *ATG2B* rs3759601 (a–f) and *ATG5* rs2245214 (g–i). Human monocytes were trained with BCG for 24 h, washed and incubated in RPMI (10% human serum) for 6 d, after which they were restimulated for 24 h with a second stimulus (LPS, *Bb*, or *C. albicans*). Proinflammatory cytokine production (IL-6 and TNF-α) was assessed by ELISA in the supernatants. (j–k) PBMCs isolated from volunteers carrying different genotypes for SNPs rs3759601 or rs2245214 were stimulated for 24 h with LPS or *B. burgdorferi*. IL-6 was measured in the supernatants by ELISA. (l) Human monocytes carrying different genotypes for SNP rs3759601 were trained with BCG for 4 h. Expression of *ATG2B* was assessed by qPCR *P<0.05, **P<0.01.

To further explore the role of autophagy in the nonspecific protection of BCG in innate immune cells, we examined the effects of genetic polymorphisms in autophagy genes for the BCG-induced trained immunity *in vitro* and *in vivo*. The genotypes of nine SNPs in eight autophagy genes were correlated with the capacity of BCG to induce trained immunity in a group of 72 volunteers. The rs3759601 *ATG2B* SNP was found to be strongly associated with trained immunity; the ability to develop training characteristics following BCG treatment was observed in monocytes isolated from individuals carrying the GG (major) or CG genotype but not in those carrying the CC (minor) genotype (plus strand coding) (Figure 2a–f). A similar effect, though less clear, was apparent for the rs2245214 *ATG5* SNP (Figure 2g–i). No significant association was found between the nonspecific protection of BCG and polymorphisms in *ATG10*, *ATG16L1*, *EREG*, *IRGM*, *LAMP3* and *WIPI* (Figure S3).

To test the possibility that the association between SNPs and differences in cytokine production of BCG-trained monocytes was due to differential intrinsic capacity of the cells to produce cytokines, we stimulated monocytes bearing different *ATG2B* (Figure 2j) or *ATG5* (Figure 2k) alleles with LPS or *B. burgdorferi* for 24 hours. We noted no differences in cytokine release, indicating that the capacity of cells to release proinflammatory cytokines upon stimulation was not responsible for the observed association between autophagy SNPs and BCG-induced trained immunity. Next to that, the effect of the rs3759601 SNP on the transcription of the *ATG2B* gene was assessed after training. We observed increased levels of *ATG2B* transcripts in BCG-trained cells of individuals carrying the GG genotype but not in those carrying the CC genotype (Figure 2l). Increased *ATG2B* levels could also be found in β-glucan trained individuals carrying the GG genotype (Figure S4a) but no difference in *ATG2B* levels could be found in the two groups after LPS stimulation (Figure S4b). The reduced expression of *ATG2B* in individuals carrying the CC genotype of the SNP upon training with BCG could indicate a role for autophagy in trained immunity since it has been shown that the ATG2 proteins are essential for the formation of autophagosomes [11].

### Autophagy is influenced by *ATG2B* single nucleotide polymorphism

**Figure 3:**
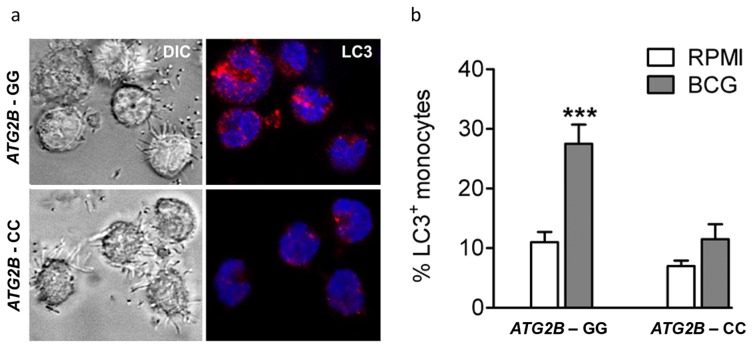
Autophagy affected by SNP in ATG2B. (a–b) Monocytes genotyped for *ATG2B* rs3759601 were seeded on coverslips, and stimulated with BCG. After 1 hour of stimulation, cells were fixed and stained with an antibody against LC3. Slides were analyzed by confocal microscopy. Data are representative for 3 experiments.

To identify the effect of rs3759601 in *ATG2B* on autophagy, the amount of LC3^+^ vesicles in BCG stimulated monocytes of individuals carrying the major or minor variant of the SNP have been compared. A decrease in autophagosome formation of individuals carrying the CC genotype can be seen as demonstrated by a lower percentage of LC3^+^ monocytes (Figure 3a–b).

### *ATG2B* single nucleotide polymorphism influences in vivo training of monocytes

**Figure 4:**
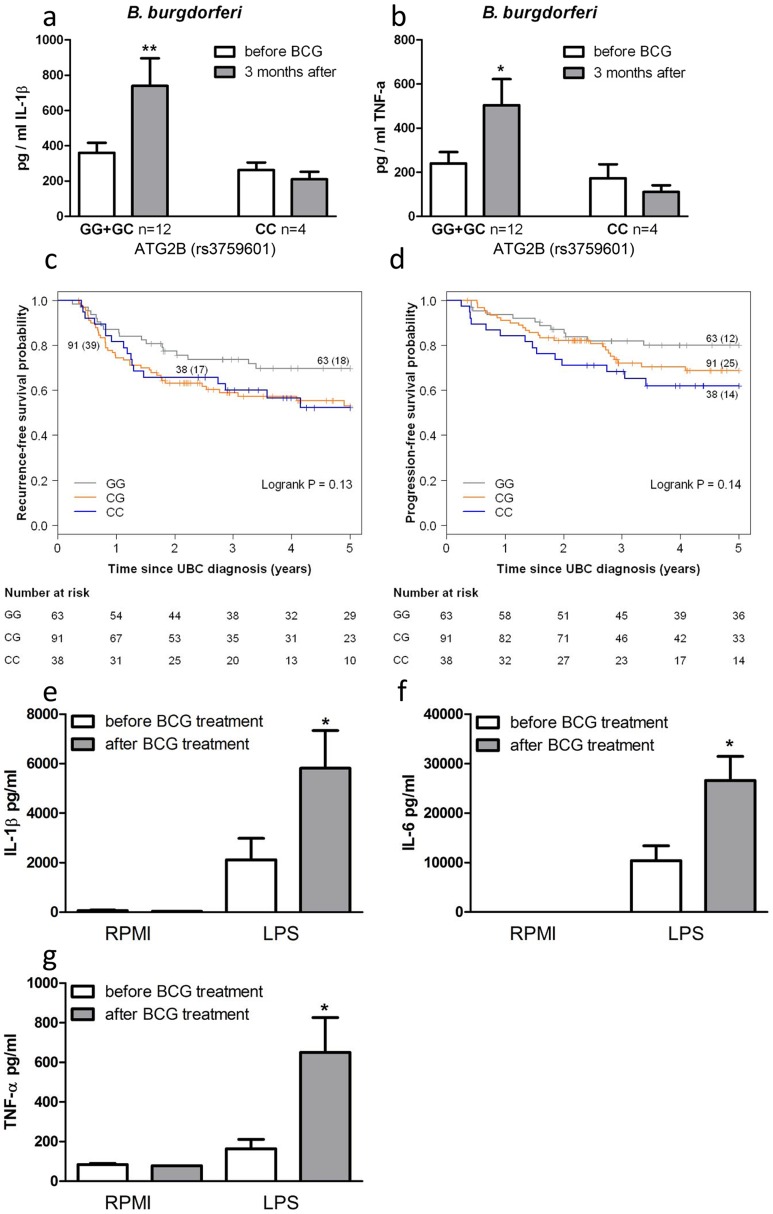
SNP in *ATG2B* affects the efficacy of *in vivo* BCG-induced trained immunity. (a–b) Monocytes isolated before and 3 months after vaccination of 16 naïve (nonexposed) volunteers were stimulated *in vitro* with *B. burgdorferi*. Proinflammatory cytokine production (IL-1β [a], TNF-α [b]) was assessed by ELISA in the supernatants. (c–d) Kaplan-Meier curves for recurrence-free (c) and progression-free (d) survival according to rs3759601 SNP genotype of 192 patients suffering from non-muscle invasive bladder cancer treated with ≥6 intravesical instillations of BCG. Each drop in a probability curve indicates one or more events in that group. Vertical lines indicate censored patients, *i.e.* those who reached the end of their follow-up without experiencing the event. Total number of patients and number of events (between brackets) per genotype category are indicated next to the corresponding curve. Numbers of patients at risk at selected time points for each genotype category are given below the plots. (e–g) Monocytes of bladder cancer patients isolated before and after 6 intravesical BCG instillations as initial treatment were stimulated *in vitro* with LPS. Proinflammatory cytokine production (IL-1β [e], IL-6 [f], TNF-α [g]) was assessed by ELISA in the supernatants *P<0.05, **P<0.01.

To corroborate the above data, we investigated BCG-induced training of monocytes *in vivo* by testing individuals carrying different *ATG2B* alleles. Monocytes were isolated from 16 healthy volunteers, before and 3 months after vaccination with BCG. Following stimulation with LPS (Figure S5a–b) or *B. burgdorferi* (Figure 4a–b), IL-1β and TNF-α production was significantly higher 3 months after vaccination in individuals who were bearing at least one G allele of the *ATG2B* SNP (n = 12), while monocytes isolated from individuals carrying the CC genotype (n = 4) showed no change in cytokine production after BCG vaccination.

### SNP in *ATG2B* correlates with the progression and recurrence of bladder cancer after BCG intravesical instillation therapy

In addition to the protective effects of BCG against secondary infections, non-specific therapy with intravesical BCG is also used as a therapeutic strategy for patients with non-muscle invasive bladder cancer (NMIBC; stages: Ta, T1, CIS) [12]. In a cohort of 192 NMIBC patients treated with at least 6 intravesical instillations of BCG we evaluated the association between the *ATG2B* SNP and prognosis in terms of recurrence and progression during the first five years after the primary NMIBC diagnosis. Analyses learned that those patients that carry one or two C alleles for *ATG2B* rs3759601 showed increased risk of recurrence (CG vs. GG: hazard ratio (HR) = 1.73 (95% confidence interval (CI): 0.99–3.03) and CC vs. GG: HR = 1.68 (95% CI: 0.78–3.27)) (Figure 4c) and progression (CG vs. GG: HR = 1.57 (95% CI: 0.79–3.12) and CC vs. GG: HR = 2.15 (95% CI: 1.00–4.66)) (Figure 4d). This finding of a correlation between the polymorphism in *ATG2B* to progression and recurrence of bladder cancer supports the hypothesis of a clinical relevance of the autophagy gene for the non-specific protective effects exerted by BCG. In addition, the responsiveness of circulating monocytes of bladder cancer patients has been investigated before and after BCG-therapy. Of high interest, individuals who received intravesical BCG therapy showed an increased cytokine response of their monocytes after stimulation with LPS *in vitro* (Figure 4e–g).

### Pharmacologic or genetic inhibition of autophagy blocks epigenetic reprogramming of monocytes in response to BCG training

**Figure 5:**
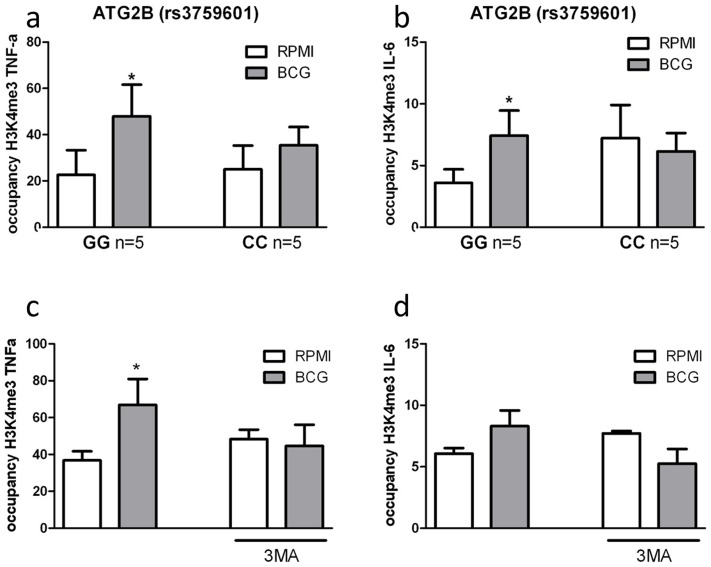
Impairment of autophagy decreases trimethylation at H3K4 in human monocytes. ChIP analysis of the enrichment of H3K4me3 at the promoter of (A) TNF-α and (B) IL-6 in human monocytes isolated from volunteers carrying the major variant (GG) or minor variant (CC) alleles for *ATG2B* after training with BCG. ChIP analysis of the enrichment of H3K4me3 at the promoter of (C) TNF-α and (D) IL-6 in human monocytes trained with BCG in the presence or absence of 3MA *p<0.05, **p<0.01.

Epigenetic reprogramming of monocytes is a crucial immunological mechanism underlying nonspecific protection by BCG. Stable changes in histone trimethylation at the level of lysine 4 of histone 3 (H3K4), a post-translational modification associated with the regulation of immune-related genes [13], is one of the mechanisms responsible for enhanced cytokine production after re-stimulation of trained monocytes [5]–[7]. Therefore, we assessed whether trimethylation of H3K4 due to nonspecific training by BCG was influenced by the *ATG2B* polymorphism or inhibition of autophagy by 3MA. Consistent with our hypothesis, H3K4 trimethylation was significantly increased at the IL-6 and TNF-α promoters in BCG-trained monocytes from volunteers bearing the *ATG2B* G allele (Figure 5a–b). In contrast, volunteers homozygous for the *ATG2B* C allele did not show any increase in trimethylation at H3K4 at the cytokine promoters after BCG-training. Furthermore, inhibition of autophagy by 3MA blocked the H3K4 trimethylation at IL-6 and TNF-α promoters in BCG-trained monocytes (Figure 5c–d), supporting the hypothesis of a central role of autophagy in the epigenetic reprogramming of monocytes induced by BCG.

## Discussion

BCG is a live attenuated vaccine which is routinely administered at birth in low-income countries, protecting newborns against disseminated tuberculosis and tuberculosis meningitis [14]. However, in addition to its specific protection against childhood tuberculosis, epidemiological studies have demonstrated that BCG protects against infant mortality independent of its effect on tuberculosis, suggesting a nonspecific protection against unrelated infections [15]–[24]. Next to that, BCG treatment has long been used as a non-specific immunostimulatory therapy in urothelial cell carcinomas [25]. Recently, these non-specific protective mechanisms of BCG have been associated with epigenetic reprogramming of innate immune cells in a process called *trained immunity* [7]. In the present study we show that autophagy is a central event modulating trained immunity induced by BCG. Moreover, polymorphisms in autophagy genes such as *ATG2B* control trained immunity in both *in vitro* and *in vivo* models, as well as the non-specific therapeutic effects of BCG in patients with bladder cancer.

An important difference has to be noted between the effect of *ATG2B* polymorphism on BCG training against secondary infections and BCG used as a treatment against non-muscle invasive bladder cancer. BCG training of monocytes against unrelated secondary infections could only be modulated by an *ATG2B* polymorphism expressed on both alleles. Heterozygote individuals were still trainable with the vaccine. On the contrary, the prognosis in terms of recurrence and progression of non-muscle invasive bladder cancer decreased with only one affected allele. The different route of BCG administration, as well as several disease-related mechanisms could be the explanation of this event. To further unravel the different mechanisms behind this phenomenon, a pilot study has been performed to investigate whether BCG installation in the bladder could induce a state of trained immunity. The cytokine response of ex-vivo stimulated monocytes of BCG treated bladder cancer patients increased in response to LPS compared to the pre-treatment response.

In addition to the aspects discussed above, there are also a few limitations of the current study. Thus, although we demonstrate the role of autophagy for BCG-induced trained immunity, additional studies are needed to decipher the precise pathway linking autophagy to the epigenetic modifications observed during trained immunity. A second important aspect is the fact that the genetic study has been performed in a relatively small cohort of patients with bladder carcinoma, and it needs to be validated by independent studies. Finally, the role of autophagy gene SNPs for the effects of BCG on infections also needs to be evaluated. The role of BCG for protection against infection is currently investigated by a large Danish study in 4500 newborn children (http://calmette-studiet.dk/), and the effect of the autophagy polymorphisms on the effects of BCG is an important aspect to be assessed.

A key question regarding trained immunity refers to the signaling and molecular mechanisms responsible for its induction. As shown previously, exposure of monoctyes to BCG induces high levels of H3K4 trimethylation at the promoter level of inflammatory genes, which correlates with long-term increased production of proinflammatory cytokines, a hallmark of trained immunity [6], [7]. Next to that, the blockage of histone acetyltransferases inhibits the training of monoctyes [26] suggesting also an important role of acetylation in trained immunity which will be further studied in the future.

The discovery that autophagy modulates trained immunity may have important consequences. It provides understanding of an important immunological process, although future studies are warranted to identify the molecular mechanisms through which autophagy mediates the epigenetic changes responsible for trained immunity. Restriction of reactive oxygen species release from damaged mitochondria, or processing of microbial ligands such as peptidoglycans [9], may represent two potential candidate mechanisms. Furthermore, identification of autophagy as a driver of trained immunity opens new possibilities for improvement of future BCG-based vaccines to be used against infections and malignancies.

## Materials and Methods

### Ethics statement

All human experiments were conducted according to the principles expressed in the Declaration of Helsinki. Before taking blood, informed written consent of each human subject was obtained. The study was approved by the review board of the department of Medicine of the Radboud University Nijmegen Medical Centre. The BCG *in vivo* study was approved by the Arnhem-Nijmegen Ethical Committee. For the NBCS, all participants gave written informed consent and the study was approved by the Institutional Review Board of the RUMC. All data analyzed were anonymized.

### Healthy volunteers

*In vitro* cytokine stimulation experiments were performed with PBMCs isolated from buffy coats obtained from healthy volunteers (Sanquin Bloodbank, Nijmegen, the Netherlands). To analyze the effect of gene polymorphisms on trained immunity, blood was drawn from a group of healthy volunteers (age 23–73). For the *in vivo* BCG model, subjects (aged 20–36) who were scheduled to receive a BCG vaccination at the public health service, due to travel or work in tuberculosis-endemic countries, were asked to participate in this trial. Blood was drawn before and 3 months after the BCG vaccination. Informed consent was obtained from all human subjects.

The bladder cancer patients included in this study were selected from a total of 1,602 patients with primary urinary bladder cancer (UBC) from the Nijmegen Bladder Cancer Study (NBCS). The NBCS served as the Dutch discovery population in the UBC genome-wide association study led by Radboud University Medical Centre (RUMC, Nijmegen, the Netherlands) and deCODE Genetics (Reykjavik, Iceland). The NBCS has been described in detail before [27]. Cases with a previous or simultaneous diagnosis of upper urinary tract cancer, based on information from the Netherlands Cancer Registry, were excluded. Detailed clinical data concerning diagnosis, stage, treatment, and disease course (tumor recurrence and progression) were collected retrospectively based on a medical file survey. In the analysis we included a total of 192 cases with non-muscle invasive bladder cancer (NMIBC; stage Ta/T1/CIS) that received at least 6 intravesical BCG instillations as initial treatment (median follow-up time from initial transurethral resection of bladder tumor until last urological check-up visit was 5.2 years (range: 0.4–20)). All patients were from Caucasian background.

### Microorganisms

*C. albicans* ATCC MYA-3573 (UC 820) yeast was heat-inactivated for 30 min at 95°C. *B. burgdorfer*i, ATCC strain 35210, was cultured at 33°C in Barbour-Stoenner-Kelley (BSK)-H medium (Sigma-Aldrich) supplemented with 6% rabbit serum. Spirochetes were grown to late-logarithmic phase and examined for motility by dark-field microscopy. Bacteria were harvested by centrifugation of the culture at 7000× g for 15 min and washed twice with sterile PBS (pH 7.4).

### Stimulation experiments

The mononuclear cell fraction was obtained by density centrifugation of blood diluted 1∶1 in pyrogen-free saline over Ficoll-Paque (Pharmacia Biotech, Pittsburgh, Pennsylvania, USA). Cells were washed twice in saline and resuspended in culture medium (RPMI; Invitrogen, Carlsbad, California, USA) supplemented with 50 mg/L gentamicin, 2 mM L-glutamine and 1 mM pyruvate. PBMCs were counted in a Coulter counter (Coulter Electronics, Brea, California, USA) and their number was adjusted to 5×10^6^ cells/ml. A total of 5×10^5^ cells in a 100 µl volume was added to round-bottom 96-well plates (Greiner) with RPMI, *E. coli* LPS (10 ng/ml) or *B. burgdorferi* (1×10^6^/ml). After 24 h, the supernatants were collected and stored at −20°C until being assayed.

For training experiments, PBMCs (5×10^5^ for cytokine analysis; 10×10^6^ for ChIP analysis) were incubated for 1 h at 37°C in 5% CO_2_. Adherent monocytes were selected by washing out nonadherent cells with warm PBS. Thereafter, cells were preincubated with RPMI, BCG vaccine (1 µg/ml BCG vaccine SSI from the Netherlands Vaccine Institute) or β-1,3-(D)-glucan (β-glucan) (10 ng/ml; kindly provided by Professor David Williams) for 24 h (4 h for Real-time PCR). After a resting period of 6 d in RPMI including 10% serum, cells were stimulated with *E. coli* LPS (10 ng/ml), *C. albicans* (1×10^6^/ml), *B. burgdorferi* (1×10^6^/ml), or RPMI for an additional 24 h. Supernatants were stored at −20°C until ELISA was performed. In the “inhibition” experiments, before training with BCG or β-glucan, the adherent monocytes were preincubated for 1 h with 10 mM 3-methyl adenine (3MA, Sigma).

### Cytokine measurements

Concentrations of human IL-1β, IL-6 and TNF-α were determined in duplicates using commercial ELISA kits (Sanquin, Amsterdam, or R&D Systems, Minneapolis), in accordance with the manufacturers' instructions.

### Real-time PCR

RNA from stimulated monocytes was isolated using TRIzol reagent (Invitrogen) according to the manufacturer's instructions. Isolated RNA was reverse-transcribed into complementary DNA using iScript cDNA synthesis kit (Bio-Rad). Quantitative real-time PCR was performed using Power SYBR Green PCR Master Mix (Applied Biosystems) using a 7300 Real-time PCR system (Applied Biosystems). In each PCR a melting curve analysis was included to control for a specific PCR amplification. Primers used for the experiments (final concentration 10 µM) are shown below. Real-time quantitative PCR data were corrected for expression of the housekeeping gene *B2M*. Human *ATG2B* forward: ACCAGAGATAGCACCTTCTGAC and reverse: CCAATTAACCGTCCAATCTG; human *B2M* forward: ATGAGTATGCCTGCCGTGTG and reverse: CCAAATGCGGCATCTTCAAAC.

### Isolation of genomic DNA and single nucleotide polymorphism analysis

*In vitro* training experiment: Using NCBI SNP database we selected SNPs in autophagy genes previously associated to diseases or with a minor allele frequency of at least 5% (*ATG10* (rs1864183), *ATG10* (rs3734114), *ATG16L1* (rs2241880), *ATG2B* (rs3759601) [allele frequency: G = 70%; C = 30%], *ATG5* (rs2245214), *EREG* (rs78803121), *IRGM* (rs4958847), *LAMP3* (rs482912), *WIPI* (rs883541)). Blood samples were obtained by venapuncture. Genomic DNA was isolated from EDTA blood using standard methods, and 5 ng of DNA was used for genotyping. Multiplex assays were designed using Mass ARRAY Designer Software (Sequenom) and genotypes were determined using Sequenom MALDI-TOF MS according to manufacturer's instructions (Sequenom Inc., San Diego, CA, USA) as described previously [28].

*In vivo* BCG-cohort: DNA was isolated using the Gentra Pure Gene Blood kit (Qiagen), in accordance with the manufacturer's protocol for whole blood. DNA was dissolved in a final volume of 100 µl buffer. Genotyping of single nucleotide polymorphisms (SNPs) was performed using a pre-designed TaqMan H SNP genotyping assay (Applied Biosystems) according to the manufacturer's protocol.

NBCS: All bladder cancer patients were genotyped using the Illumina Infinium HumanCNV370-duo Bead-Chips. Imputation was performed (IMPUTE version 2.1 software) using the 1000 Genomes low-coverage pilot haplotypes (released June 2010, 120 chromosomes) and the HapMap3 haplotypes (released February 2009, 1920 chromosomes) as a combined reference panel [27]. SNP rs3759601 was imputed with IMPUTE info_score 0.99. The SNP followed Hardy-Weinberg equilibrium.

### Transcriptome analysis

Gene expression was performed as described previously [29] and assessed using Illumina Human HT-12 Expression BeadChip according to manufacturer's instructions. The Illumina LIMS platform, BeadStudio was employed to perform image analysis, bead-level processing, and quantile normalization of array data.

### Chromatin immunoprecipitation

Adherent monocytes were cultured as described above (see *Stimulation Experiments*). ChIP was performed using antibodies against H3K4me3 (Diagenode). ChIPed DNA was processed further for qPCR analysis. The following primers were used in the reaction (5′-3′): TNF-α forward: CAGGCAGGTTCTCTTCCTCT, TNF-α reverse: GCTTTCAGTGCTCATGGTGT; IL-6 forward: TCGTGCATGACTTCAGCTTT, IL-6 reverse: GCGCTAAGAAGCAGAACCAC; myoglobin forward: AGCATGGTGCCACTGTGCT, myoglobin reverse: GGCTTAATCTCTGCCTCATGAT.

### Immunofluorescence staining

For immunofluorescence imaging, monocytes were seeded on coverslips pretreated with polylysine, fixed with 4% PFA for 15 min at room temperature followed by 10 min of fixation with ice-cold methanol at −20°C. After two washing steps with PBS, cells were permeabilized by 0.1% saponin (Sigma-Aldrich), blocked for 30 min in PBS plus 2% BSA, incubated for 1 h with a mouse mAb to LC3 (1∶50; Nanotools), washed twice in PBS plus 2% BSA and stained by a secondary Alexa Fluor 555 goat anti-mouse Ab (1∶500; Molecular Probes), followed by DNA staining with 10 µM TO-PRO-3 iodide (642/661; Invitrogen). After the washing steps, slides were mounted in Prolong Gold antifade media (Molecular Probes). Images were acquired using a laser-scanning spectral confocal microscope (TCS SP2; Leica Microsystems) and LCS Lite software (Leica microsystems). 2 fields/donor including at least 40 cells each were counted and compared for the amount of LC3.

### Statistical analysis

Data are expressed as mean ± SEM unless mentioned otherwise. Differences between experimental groups were tested using the non-parametrical two-sided Mann-Whitney *U* test (no normal distribution of measured cytokines); differences between multiple time points within one group (before versus after treatment) were tested using the Wilcoxon matched pair test (unless stated otherwise) performed on GraphPad Prism 4.0 software (GraphPad). *P* values of ≤0.05 were considered statistically significant.

Kaplan-Meier survival and Cox proportional hazard regression analyses were performed to evaluate the association between rs3759601 and recurrence- and progression-free survival. Log-rank tests were calculated to compare survival curves between genotype categories. Imputed genotype probabilities were transformed to hard genotype calls based on a probability threshold of >0.90. Statistical analyses were performed using IBM SPSS Statistics for Windows 20 (IBM Corp., Armonk, NY, USA) and survival plots were drawn using R software v3.0.2 (package ‘survival’) (R Development Core Team, Vienna, Austria).

## Supporting Information

Figure S1 Role of autophagy for the training of monocytes. BCG (a–b, e–f) or β-glucan (c–d, g–h) training *in vitro* in the presence or absence of 3MA or wortmannin using freshly isolated human monocytes and different stimuli for restimulation (LPS, *B. burgdorferi*). TNF-α (a–d) and IL-6 (e–h) were assessed by ELISA in the supernatants. *P<0.05, **P<0.01.
(TIF)

Figure S2 Viability of monocytes after chemical blocking of autophagy for 24 h. BCG or β-glucan training *in vitro* in the presence or absence of 3MA or wortmannin using freshly isolated human monocytes. Cell viability tested by CytoTox 96 NonRadioactive Cytotoxicity Assay after 24 h (a), 3 days (b) and 6 days (c).
(TIF)

Figure S3 Polymorphisms in *ATG10, ATG16L1, EREG, IRGM, LAMP3 and ATG18* do not diminish the training capacity of human monocytes. Blood was collected from volunteers and genotyped for *ATG10* rs1864183 and rs3734114 (a–d), *ATG16L1* rs2241880 (e–f), *EREG* rs78803121 (g–h), *IRGM* rs4958847 (i–j), *LAMP3* rs482912 (k–l) *and ATG18* rs8835411 (m–n). Human monocytes were trained with BCG for 24 h, washed and incubated in RPMI (10% human serum) for 6 d, after which they were restimulated for 24 h with a second stimulus (LPS or *Bb*). Proinflammatory cytokine production (TNF-α) was assessed by ELISA in the supernatants.
(TIF)

Figure S4 SNP in *ATG2B* affects its expression after training but not stimulation. Human monocytes carrying different genotypes for SNP rs3759601 were trained with β-glucan [a] or stimulated with LPS [b] for 4 h. Expression of *ATG2B* was assessed by qPCR.
(TIF)

Figure S5 SNP in *ATG2B* affects the efficacy of *in vivo* BCG-induced trained immunity. Monocytes isolated before and 3 months after vaccination of 16 naïve (nonexposed) volunteers were stimulated *in vitro* with LPS. Proinflammatory cytokine production (IL-1β [a], TNF-α [b]) was assessed by ELISA in the supernatants.
(TIF)
