# Supplementary figures and images for: Autophagy Controls BCG-Induced Trained Immunity and the Response to Intravesical BCG Therapy for Bladder Cancer
Source: PLoS Pathog. 2014 Oct 30;10(10):e1004485. doi: 10.1371/journal.ppat.1004485 (PMC4214925; doi:10.1371/journal.ppat.1004485)

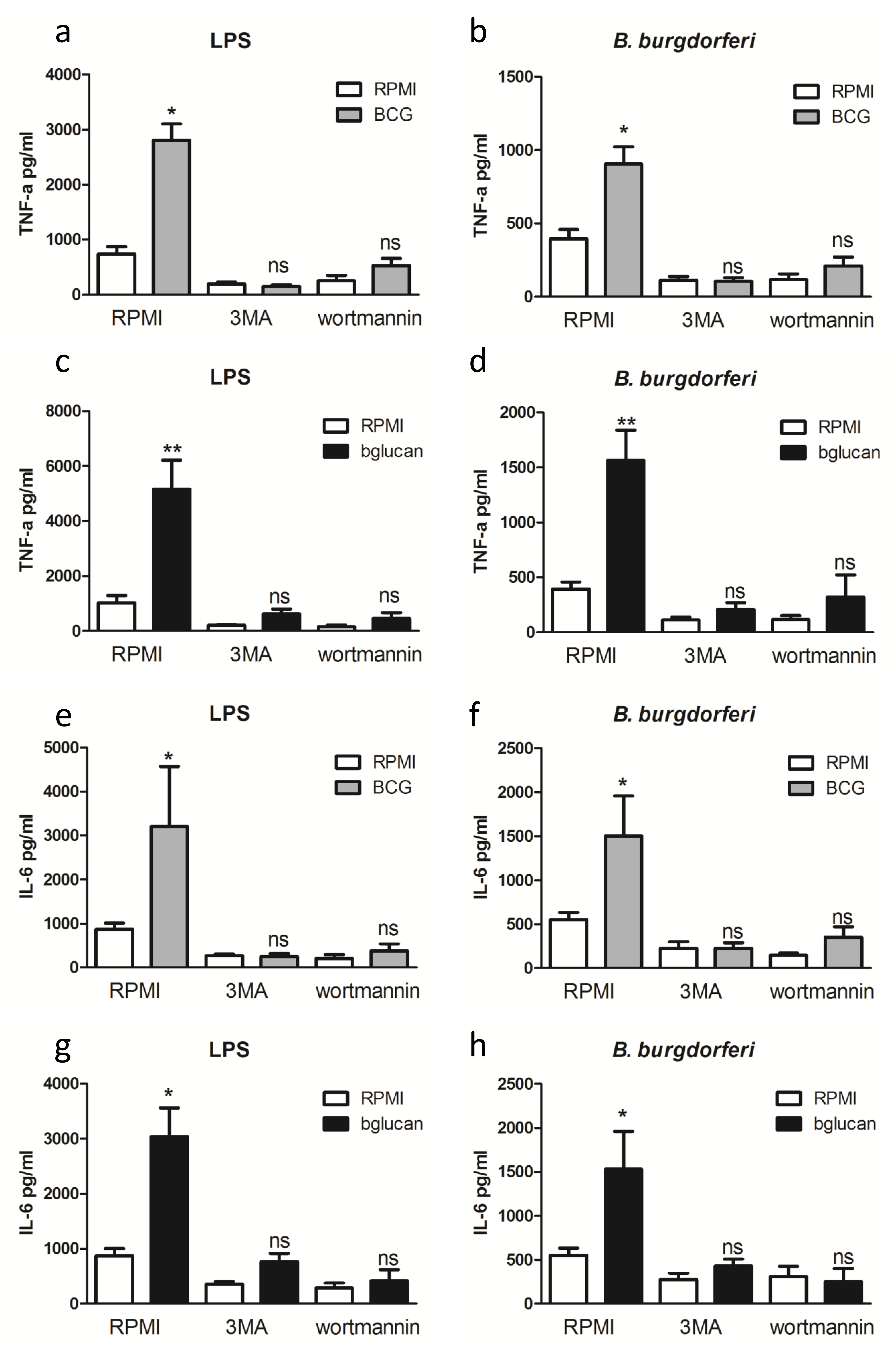

Supplement: Figure S1 — Role of autophagy for the training of monocytes. BCG (a–b, e–f) or β-glucan (c–d, g–h) training in vitro in the presence or absence of 3MA or wortmannin using freshly isolated human monocytes and different stimuli for restimulation (LPS, B. burgdorferi). TNF-α (a–d) and IL-6 (e–h) were assessed by ELISA in the supernatants. *P<0.05, **P<0.01. (TIF) [file ppat.1004485.s001.tif]

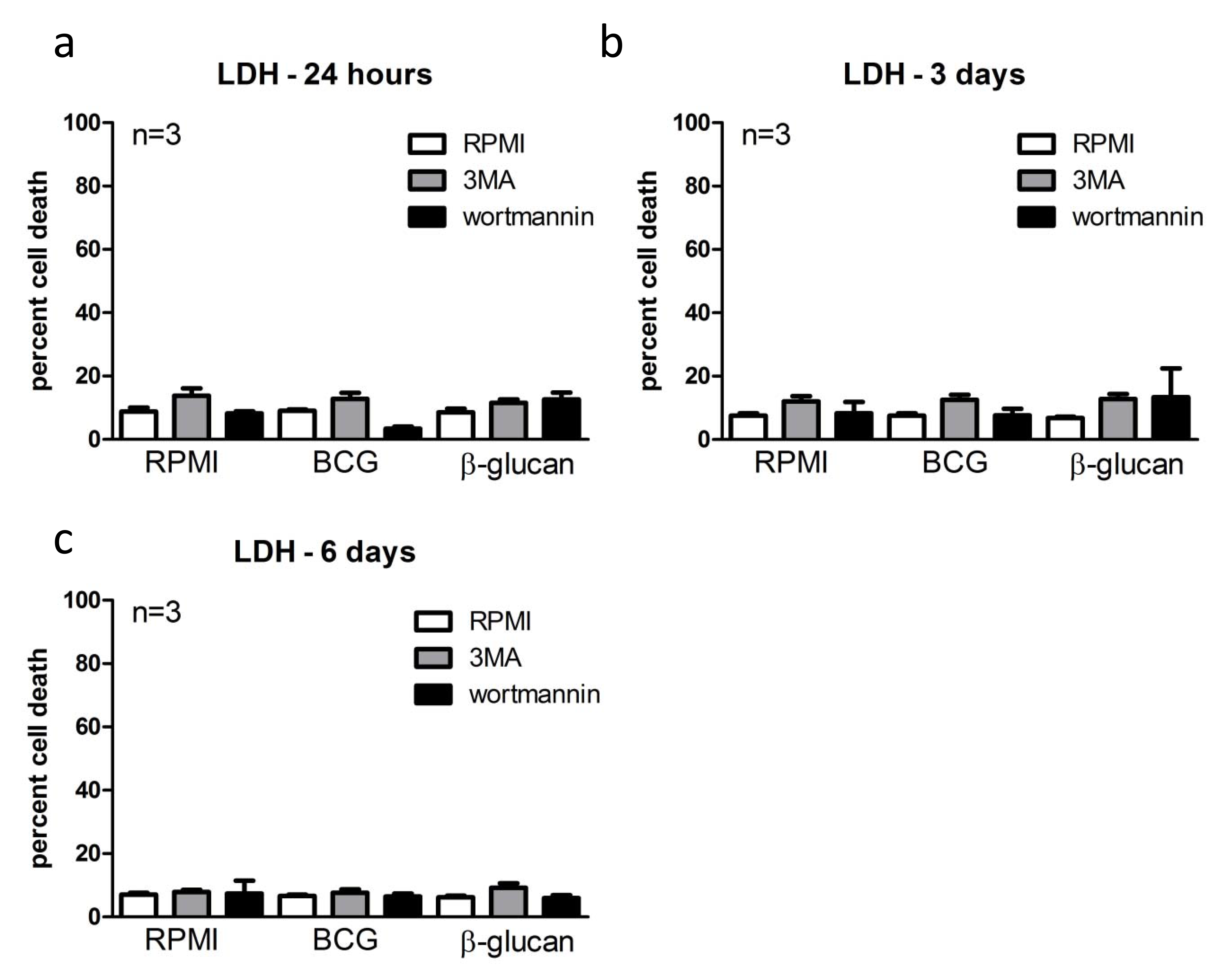

Supplement: Figure S2 — Viability of monocytes after chemical blocking of autophagy for 24 h. BCG or β-glucan training in vitro in the presence or absence of 3MA or wortmannin using freshly isolated human monocytes. Cell viability tested by CytoTox 96 NonRadioactive Cytotoxicity Assay after 24 h (a), 3 days (b) and 6 days (c). (TIF) [file ppat.1004485.s002.tif]

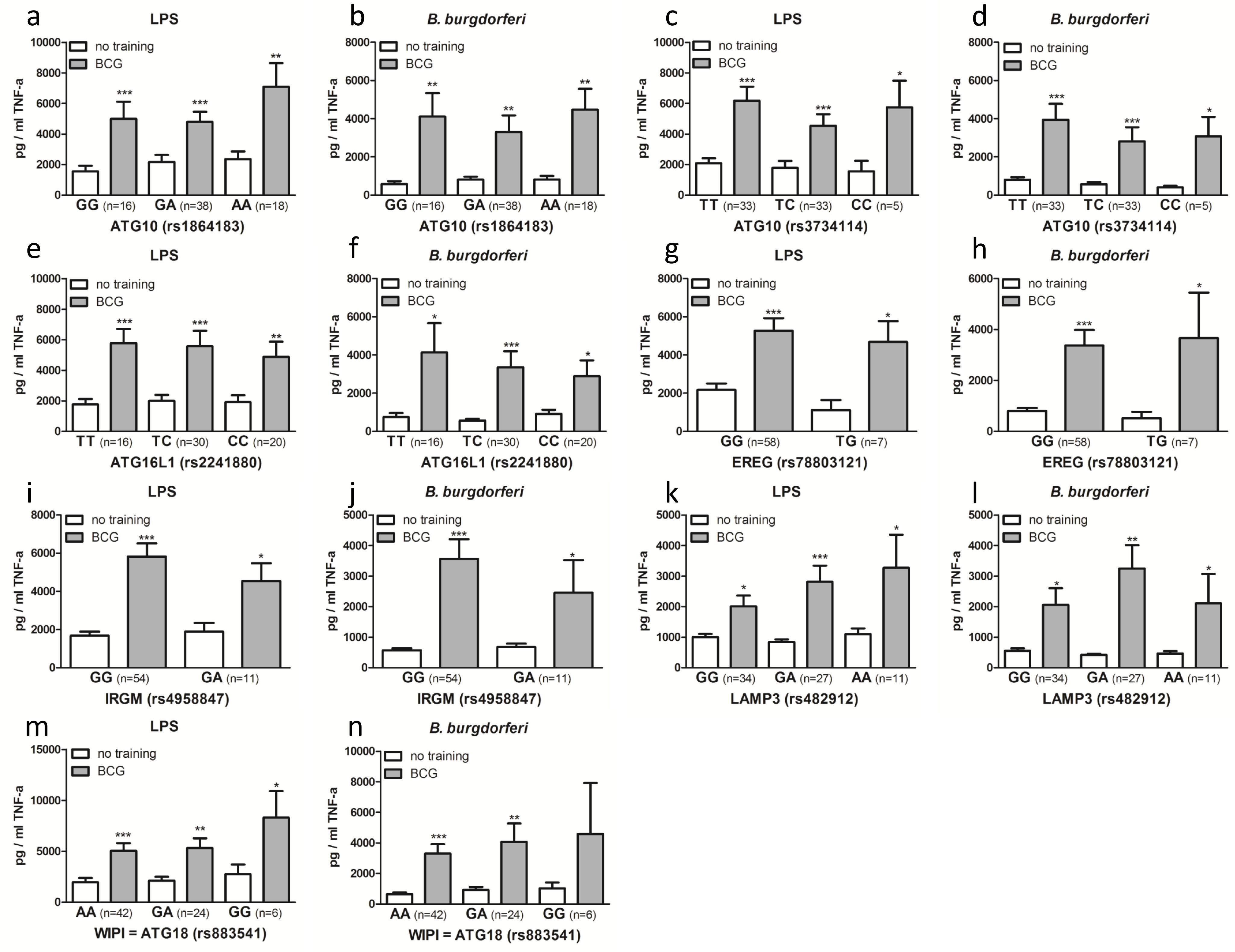

Supplement: Figure S3 — Polymorphisms in ATG10, ATG16L1, EREG, IRGM, LAMP3 and ATG18 do not diminish the training capacity of human monocytes. Blood was collected from volunteers and genotyped for ATG10 rs1864183 and rs3734114 (a–d), ATG16L1 rs2241880 (e–f), EREG rs78803121 (g–h), IRGM rs4958847 (i–j), LAMP3 rs482912 (k–l) and ATG18 rs8835411 (m–n). Human monocytes were trained with BCG for 24 h, washed and incubated in RPMI (10% human serum) for 6 d, after which they were restimulated for 24 h with a second stimulus (LPS or Bb). Proinflammatory cytokine production (TNF-α) was assessed by ELISA in the supernatants. (TIF) [file ppat.1004485.s003.tif]

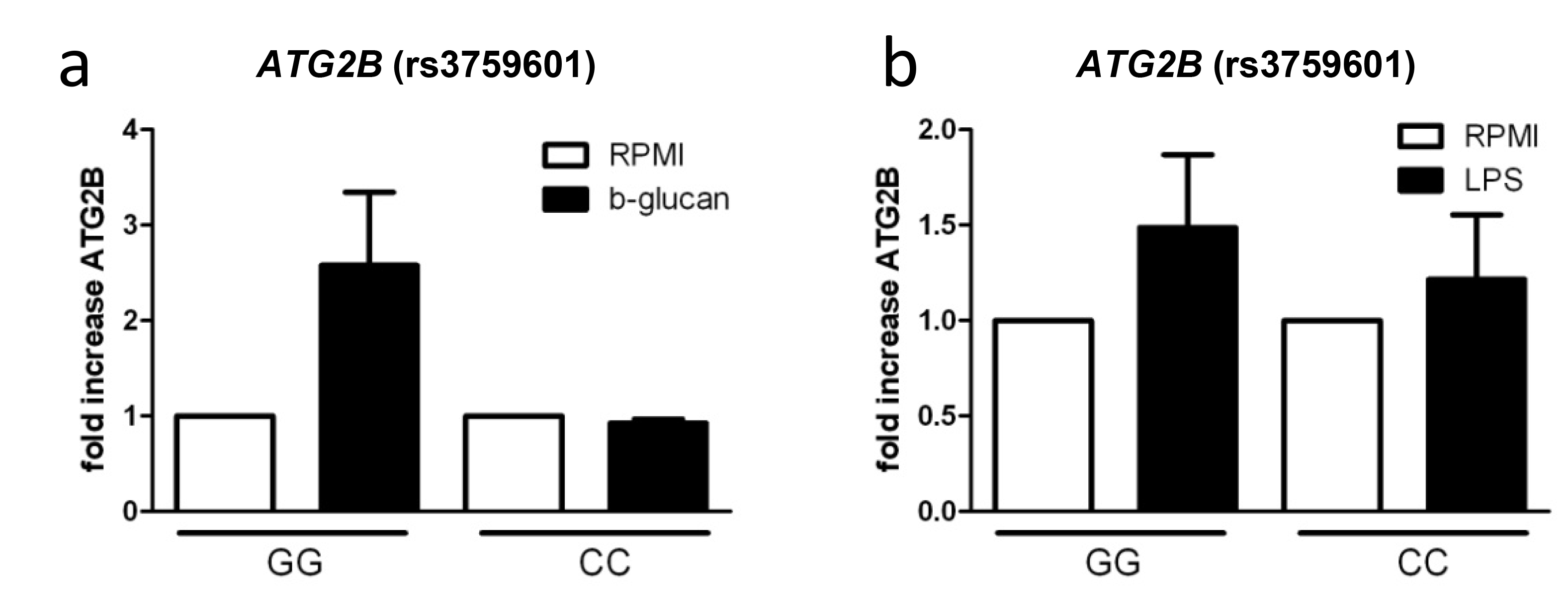

Supplement: Figure S4 — SNP in ATG2B affects its expression after training but not stimulation. Human monocytes carrying different genotypes for SNP rs3759601 were trained with β-glucan [a] or stimulated with LPS [b] for 4 h. Expression of ATG2B was assessed by qPCR. (TIF) [file ppat.1004485.s004.tif]

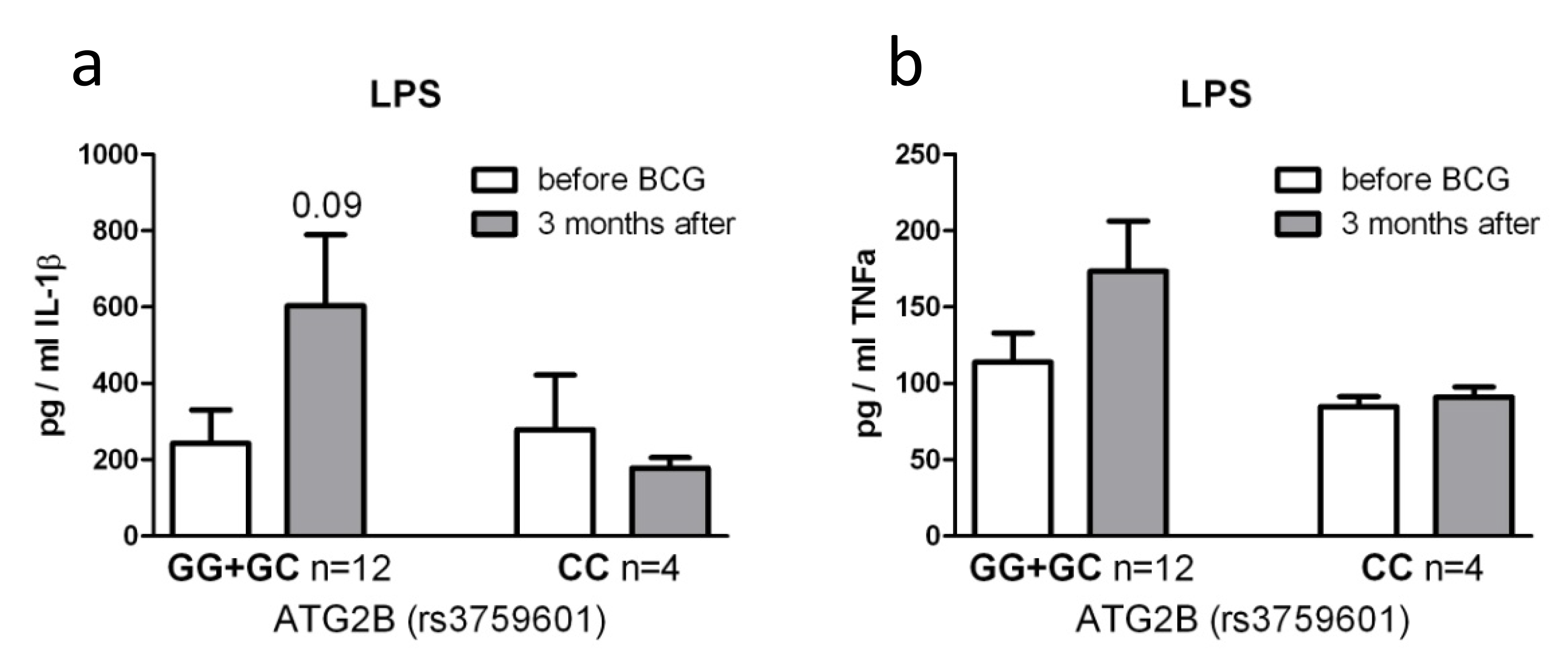

Supplement: Figure S5 — SNP in ATG2B affects the efficacy of in vivo BCG-induced trained immunity. Monocytes isolated before and 3 months after vaccination of 16 naïve (nonexposed) volunteers were stimulated in vitro with LPS. Proinflammatory cytokine production (IL-1β [a], TNF-α [b]) was assessed by ELISA in the supernatants. (TIF) [file ppat.1004485.s005.tif]
